# Impacts of intimate partner violence and sexual abuse on antiretroviral adherence among adolescents living with HIV in South Africa

**DOI:** 10.1097/QAD.0000000000003440

**Published:** 2022-12-05

**Authors:** Lucie D. Cluver, Siyanai Zhou, Mark Orkin, William Rudgard, Franziska Meinck, Nontokozo Langwenya, Marissa Vicari, Olanrewaju Edun, Lorraine Sherr, Elona Toska

**Affiliations:** a Department of Social Policy and Intervention, University of Oxford, Oxford, UK; b Department of Psychiatry and Mental Health and Centre for Social Science Research; c Centre for Social Sciences Research, Faculty of Humanities; d Division of Socio-Behavioural Sciences, School of Public Health and Family Medicine, Faculty of Health Sciences, University of Cape Town, Cape Town; e Wits/Medical Research Council Development Pathways to Health Research Unit, School of Clinical Medicine, University of the Witwatersrand, Johannesburg, South Africa; f School of Social and Political Science, University of Edinburgh, Scotland, United Kingdom; g School of Public Health, University of the Witwatersrand, Johannesburg; h North-West University, Optentia Research Focus Area, Vanderbijlpark, South Africa; i International AIDS Society, Geneva, Switzerland; j MRC Centre for Global Infectious Disease Analysis, School of Public Health, Imperial College London; k Institute for Global Health, University College London, London, UK; l Faculty of Humanities, Department of Sociology, University of Cape Town, Cape Town, South Africa.

**Keywords:** adherence, adolescents, HIV, intimate partner violence, sexual abuse, South Africa

## Abstract

We are failing to reach 95–95–95 for adolescents living with HIV (ALHIV). Sexual abuse and intimate partner violence (IPV) may impact antiretroviral therapy (ART) adherence, with high rates of 17.4 and 29.7%, respectively, across the southern sub-Saharan African region. However, evidence on their associations with adolescent ART adherence remains limited, with only three cross-sectional studies globally.

**Design:**

A prospective cohort of ALHIV (sample *N* = 980, 55% female individuals, baseline mean age 13.6 years) were recruited from 53 health facilities in South Africa's Eastern Cape Province and responded to a structured questionnaire at 18-month and 36-month follow-up (2015–2016, 2017–2018).

**Methods:**

A repeated-measures random effects model assessed multivariable associations of self-reported sexual abuse and IPV with past-week ART adherence, controlling for individual, socioeconomic, and HIV-related factors. Past-week adherence was defined based on currently taking ART and not having missed any doses in the past 7 days (including weekends). We further fitted a moderation model by sex.

**Results:**

Fifty-one percent of adolescents reported consistent ART adherence at both time points. Exposure to IPV was associated with lower odds of self-reported ART adherence (aOR 0.39, 95% CI 0.21–0.72, *P* = 0.003), as was sexual abuse (aOR 0.54, 95% CI 0.29-0.99, *P* = 0.048). The marginal predicted probability of ART adherence for adolescents with no exposure to either IPV or sexual abuse was 72% (95% CI 70–74%) compared with 38% (95% CI 20–56%) for adolescents with exposure to both IPV and sexual abuse. Moderation results showed similar associations between sexual violence and ART adherence by sex.

**Conclusion:**

Sexual violence prevention and postviolence care may be essential components of supporting adolescent ART adherence. Integration of HIV and violence prevention services will require accessible services and simple referral systems.

## Introduction

We are manifestly failing to reach 95–95–95 for the world's 1.7 million adolescents living with HIV (ALHIV), of whom 91% live in sub-Saharan Africa (SSA) [1]. In this region, rates of adherence to antiretroviral treatment (ART) are lower for ALHIV than in other age groups, with very limited evidence of effective interventions. Studies show that in SSA, adolescents are approximately 50% less likely than adults to maintain adherence [1,2]. It is essential that we identify barriers to adolescent ART adherence – such as stigma, poverty, and family violence [3,4] – in order to deliver effective and targeted services. Adolescents in SSA are exposed to some of the world's highest rates of violence, over half are subjected to physical, emotional, or sexual violence from intimate and nonintimate relationships [5], with reported sharp rises in sexual abuse and intimate partner violence (IPV) because of the coronavirus disease 2019 (COVID-19) pandemic [6]. IPV is often defined as behaviour within an intimate relationship that causes physical, psychological, or sexual harm to those within the relationship [7]. Estimates on the prevalence of lifetime IPV among women ages 15–49 range between 22 and 33%, with the prevalence of past-year IPV at approximately 14% in Southern Africa [8,9]. Sexual violence is often defined as nonconsensual completed or attempted sexual contact, or acts of a sexual nature not involving contact [10]. In this region, rates of sexual abuse were estimated at 17.4% (11.4–23.3) [11]. These overlapping forms of violence are amongst the ‘adverse childhood experiences’ (ACEs) that have been shown to have long-term health impacts, including reduced adherence to medication [12]. Amongst both girls and boys, exposure to sexual abuse and IPV have been shown to increase mental health distress and to reduce freedom and access to health services [13], all suggesting potential to impair adolescents’ ART adherence.

Despite this, we have very limited evidence that examines the impacts of sexual abuse and IPV on adolescent ART adherence. A recent systematic review (2021) of treatment adherence in adolescents with histories of ACEs [14] found one article examining the effects of IPV: a cross-sectional study of 129 perinatally infected female youth (aged 13–24 years) in South Africa [15]. This important first study found that a combined measure of past-year physical and/or sexual IPV was associated with higher odds of poor ART adherence [odds ratio (OR) = 5.37; 95% confidence interval (CI) 1.37–21.08]. Two recent studies add to the evidence base. A recent cross-sectional study with 272 youth (aged 15–24 years) in Zambia found no associations in the full sample between viral load and past-year IPV or forced sex by anyone including, but not limited to, a romantic partner. However, disaggregated by gender, this study found that for female youth, experiencing IPV was associated with higher odds of viral failure [adjusted OR (aOR) 2.28, 95% CI 1.03–5.04] [16]. In South Africa, a cross-sectional study of 1118 adolescent girls and young women living with HIV (aged 12–24 years) found no association between IPV and the presence of any ART in the blood (vs. self-report) but did find that IPV was associated with lower odds of viral suppression (aOR = 0.37, 0.18–0.75) [17].

There is more evidence amongst adult women: a 2015 meta-analysis of 13 cross-sectional studies found that IPV was associated with lower self-reported ART adherence and viral load suppression [18], with this finding supported by a scoping review in 2019 [19]. Since these reviews, associations between IPV and nonadherence have been found in cross-sectional studies of adult women in Kenya [20,21], Uganda [22], and longitudinally in South Africa [23].

There is a need for large-scale evidence to examine associations of sexual abuse and IPV with adolescent ART adherence. It is important to examine impacts for both adolescent girls and boys, and to add to the three existing cross-sectional studies with longitudinal evidence to assess whether sexual abuse and IPV exposure predict ART nonadherence. This study uses data from a prospective cohort of 1000 adolescents living with HIV in South Africa. We used standardized questionnaires and clinical records to assess the impact of sexual abuse and IPV on past-week ART adherence, validated against viral load.

## Methods

### Sample and location

This study is based on a three-wave cohort study [24] in South Africa's Eastern Cape, a province characterized by high HIV prevalence, poor infrastructure, and overburdened health systems. In 2014–2015, within one health district, we visited 53 health facilities providing ART to a minimum of five adolescents living with HIV and used clinic records to identify all adolescents (aged 10–19 years) who had ever initiated HIV care. We recruited from all of these adolescents (90% uptake), and to reach both those retained in healthcare, and those who had disengaged from care or were ‘lost to follow-up’, all adolescents identified in these clinic records were traced to their 180 communities and completed the study questionnaire at their home or a location of their choice. We also administered the questionnaire to their adolescent peers not living with HIV co-resident or in neighbouring households, to avoid the risk of stigma (*n* = 473, not included in this study).

Standardized questionnaires were developed with input from a Teen Advisory Group, prepiloted at baseline, and designed to be engaging and nonstigmatizing. The questionnaires were administered in isiXhosa or English, as chosen by the adolescent, by trained research assistants. At baseline, 1046 adolescents living with HIV completed the questionnaire, and at 12–18 months and at 24–36 months postbaseline, all adolescents who had given consent were re-approached for follow-up, respectively. The cohort had 94% retention at 12–18 months follow-up (2016–2017), and 97% retention at 3-year follow-up (2017-2018), with 3.4% mortality between baseline and second follow-up. Uptake and retention were supported by extensive training of local research assistants in empathetic engagement with adolescents, avoiding stigma by also interviewing neighbouring adolescents, and active discussion and feedback to participants on policy impacts of the study. This study uses data for 980 adolescents who completed the questionnaire at wave two and wave three of the study when IPV was assessed.

### Medical record review

At each of the 53 healthcare facilities, medical records (paper-based and electronic) were searched for every study participant. Data were extracted in two rounds using a standardized form, covering records from the baseline (2014-2015) and follow-up (2016–2017) time points of the study respectively, and included viral load results. The medical record data was further supplemented by routine pathology test data from 2014 to 2019 using the National Health Laboratory Services (NHLS) data warehouse, which archives all routinely collected laboratory data from South Africa's National HIV Programme. Demographic information (name, surname, sex, and date of birth) for adolescents in the main study who were accessing public sector HIV care and treatment, was used to link to pathology test records in the NHLS data warehouse and to extract adolescents’ records for HIV viral load. Given the absence of a unique identifier within the healthcare service, not all participants’ viral load records in the NHLS data warehouse could be retrieved. The record linkage process was at the national level, which allowed laboratory tests performed on participants data at facilities outside the study's geographic area to be included.

### Ethics and informed consent

Ethical approvals were given by the University of Cape Town (UCT/CSSR/2013/4) and (UCT/CSSR/2019/01), Oxford University (Oxford/CUREC2/12-21), provincial Departments of Health and Education, and ethical review boards of participating healthcare facilities. Findings from the studies are reported back to stakeholders within the research geographic areas as part of ongoing local knowledge-sharing. Voluntary informed consent was obtained from adolescents and also from their caregiver at any visit when an adolescent was aged less than 18 years. Confidentiality was maintained except in cases of disclosure of risk of harm. Research assistants received 1–2 months in-person training on conducting research with vulnerable children, which included training focused on violence victimization, creating a safe space during the interview, acknowledging disclosure of harmful and traumatizing events, as well as supporting adolescents with uptake of referrals, if consenting. Research assistants receive formal refresher training annually, with ongoing weekly debriefing and reflection space. Where participants reported recent abuse, rape, suicidal attempt, or other risk of significant harm, referrals were made to child protection and health services (*n* = 157 referrals made). Additionally, the research team included a registered child protection social worker (one of the Principal Investigators) who oversaw timely completion of referrals, liaised with social services, NGOs and police wherever necessary, and held weekly meetings to review referral case management. In cases where the participant declined the referral, they were encouraged to contact the research team when ready to be referred. Referral cases were noted as social harms and included in the annual study progression ethics documents.

### Measures

All outcomes, predictors, and covariates used identical measures at both time points.

#### Outcomes

The main outcome variable was defined as self-reported ART adherence in the past 7 days adherence (including weekdays and weekends), based on currently taking ART and not having missed any doses in the past 7 days [25]. Self-reported ART adherence was defined as a binary variable coded 1 (for adherence) in case adherence in the past week exceeded 95%, and coded 0 (nonadherence) if the respondent reported having taken less than all required doses in the past 3 days, having missed at least one dose in the past week, having missed at least one dose on the past weekend, or being currently not on ART (i.e. defaulting) [26,27]. The items used to define this outcome were adapted from the Patient Medication Adherence Questionnaire [28]. Viral suppression was defined based on a viral load test of 50 copies of HIV-RNA/ml or less

#### Predictors

IPV was measured using items adapted from the IPV instrument of the WHO Multi-Country Study questionnaire [29]. This questionnaire has been used in several countries, including in South Africa [23,30]. Participants were asked questions for the past 12 months such as, ‘How often did your boyfriend/girlfriend insult, swore or say something to spite (hurt) you?’, with responses to the questions scored on a five-point Likert scale. Past-year IPV was defined if the participant score was two or more on any of the items: exposure to physical IPV (e.g. pushed, shoved, grabbed, or slapped), emotional IPV (e.g., insulted, swore, or said something to spite me), and controlling behaviour (e.g., always want to know where you are) [30]. Sexual abuse by anyone was defined as past 12-month exposure to forced sex (‘Has anyone had sex with you when you did not want them to?’), contact abuse (‘Has anyone touched your private parts, or made you touch theirs, or tried to?’), and noncontact abuse (‘Has anyone made you look at their private parts or wanted to look at yours?’) [31]. Responses to the items were scored on a five-point Likert scale, and sexual abuse was defined if the participant reported any experience.

Covariates and sociodemographic controls (10 variables) were included in all regression models. Poverty was defined as a score combination of access to eight socially perceived necessities for children, as validated in a nationally representative survey [32]. Higher scores reflect less poverty. Food insecurity was measured, based on the number of days the participant reported not having enough food in the past week, using national survey measures [32]. We also measured caregiver type [24], and vertical/recent HIV acquisition [33]. Time on ART treatment (in years) was calculated based on the ART start date. Relationship status was measured based on currently having a boyfriend or girlfriend. Healthcare-related measures included dichotomies on the type of facility where the adolescents accessed care, namely: primary clinic, community health centre, or hospital. We also controlled for self-reported pill burden. Participants’ sex and mode of HIV acquisition (vertical/recent) were modelled as time-invariant variables, while the main predictors and all other covariates were modelled as time-variant.

### Statistical analysis

This analysis utilizes the random-effects framework described in the Bell *et al.*[34] study, given the repeated measure design of the data where individuals *i* (level 2) are measured on multiple occasions *t* = 2 (level 1). Eq. (1) shows a commonly used model for repeated measures individual data, the Hybrid (within-between) model for a dichotomous outcome *y*_*it*_ variable as described in [35,36]:


(1) $$\log\left(\frac{\mathrm{Pr}(y_{it}=1)}{1-\mathrm{Pr}(y_{it}=1)}\right)=\beta_{0}+\beta_{1\text{within}}(x_{it}-{\bar{x}}_{i})+\beta_{1\text{between}}{\bar{x}}_{i}+\cdots +\beta_{2}z_{i}+v_{i0}+v_{i1}(x_{it}-{\bar{x}}_{i})+e_{it0}$$

where *y*_*it*_ is the outcome variable, *x*_*it*_ is the time-varying independent variable (which is divided into two: *β*_1within_ and *β*_1between_ estimates), and *z*_*i*_ is a time-invariant independent variable. ${\bar{x}}_{i}$ represents the mean of the time-varying independent variable for each individual. The random part of the model constitutes *v*_*i*0_, which is the intercept random-effect and *v*_*i*1_, which is the within-slope random slope, and both are assumed to be normally distributed.

This model separates the within and between effects and explicitly models the heterogeneous effect of predictor variables at the individual level. Based on recommendations from Bell *et al.*[34], we can use the Wald test to test if *β*_1within_ = *β*_1between_, that is if there is no statistical difference between the two estimates [37]. If there is sufficient evidence to support this hypothesis, then one can estimate a random-effects model that does not separate between and within effects as shown below:


(2) $$\log\left(\frac{\mathrm{Pr}(y_{it}=1)}{1-\mathrm{Pr}(y_{it}=1)}\right)=\beta_{0}+\beta_{1RE}(x_{it})+\cdots +\beta_{2RE}z_{i}+(v_{i}+e_{it})$$

The benefit of combining the between and within estimates is that this is the most efficient estimator. However, if there is a significant difference between the two estimates, then the model in Eq. (1) is preferred.

#### Analysis steps

We used five steps in Stata v.16 (STATA Corp, College Station, Texas, USA). First, we assessed for any differences in the participants included in this study and those lost to study follow-up, and for complete cases; we then examined frequencies of outcome, predictors, and covariates and report *P* values (*χ*^2^) of any differences between time points. Second, we assessed for any differences between participants with viral load and those missing viral load, and we then evaluated the association between self-reported adherence and viral suppression (≤ 50 copies/ml) using available viral load records. Third, in order to proceed to the next step, we then used the Wald test to examine if there is a statistical difference between within and between effects; using Equation (1), we fitted a multivariable hybrid model to examine multivariable relationships between IPV and sexual abuse, and past-week ART adherence adjusted for all covariates (see Table S5). The Wald test results showed no sufficient evidence to justify that the within and between effects for each of the predictors are significantly different. Table S6 in the Appendix shows the goodness-of-fit test results, and the random effects model better fits the data than the variants of the Hybrid model [38]. Fourth, based on the findings from the Wald tests, we examined univariable and multivariable associations of sexual violence (IPV and sexual abuse) and covariates with past-week adherence using the random-intercept model.

The random effects model described in Equation (2), was used to utilize the repeated measures structure of the data (data from the same subjects at two time-points) as well as include time-invariant factors. For our main models, we applied the Hosmer and Lemeshow procedure [39] to select variables for inclusion in the final model (if *P* < 0.10 variables were carried forward), and all the models were fitted with robust-clustered standard errors at the individual level.

Fifth, we calculated adjusted predicted probabilities to compare probabilities of ART adherence for different combinations of sexual abuse and IPV exposure. To achieve this, we used the margins and lincom commands in Stata. Lastly, we carried out three types of sensitivity analysis namely: sex moderation analyses for the main model, model the primary predictors among only those who report being partnered in the past year controlling for covariates, and model the primary predictors on viral load as a clinic marker among those with viral load.

## Results

### Descriptive statistics

Summary statistics of the analytic sample are shown in Table 1. In this sample (*n* = 980), 57% were female individuals, and 23.9% had recently acquired HIV. Twenty-five percent lived in rural areas, and 14% lived in informal settlements at both time points. On average, participants had access to five socially perceived basic necessities, and were food insecure for less than 1 day at both time points. 41.2% lived in households where a biological parent was their main caregiver. Within the sample, 11% of adolescents reported exposure to either sexual abuse or IPV within a 2-year period, and 36.9% reported lifetime exposure. At the second time point, 3.5% of adolescents experienced IPV with the majority (76.5%) of these being female individuals, and a similar trend was observed at the third time point. Sexual abuse in the past year was reported by 49 (5%) of the adolescents at the second time point and by 1% at the third. A description of baseline characteristics of the baseline full sample, by past-week adherence in the main study, is shown in Table S1. We compared 980 participants included (complete cases) and 50 participants not included (lost-to-study follow-up), and there were no differences on most baseline characteristics, but participants lost-to-study follow-up were more likely to be older (see Table S2).

**Table 1:** Descriptive summary statistics over the two-time points (*N* = 980).

	Timepoint 2 (*N* = 980)	Timepoint 3 (*N* = 980)	
Variables	*N* (%)	*N* (%)	*P* value^a^
Sociodemographic factors			
Female	556 (57.0)	556 (57.0)	1.00
Age (mean/SD)	15.25 (3.03)	16.42 (3.02)	**<0.001**
Rural residence	244 (25.0)	237 (24.2)	0.71
Poverty (mean/SD)	5.52 (2.28)	5.66 (2.29)	0.17
Food insecurity (mean/SD)	0.52 (1.04)	0.42 (0.93)	**0.031**
Caregiver biological parent	404 (41.2)	404 (41.2)	1.00
In a relationship (currently)	301 (30.7)	322 (33.1)	0.266
Other covariates			
Health facility (Community health centre)	76 (7.8)	168 (17.1)	**<0.001**
Health facility (Hospital)	220 (22.5)	147 (15.0)	**<0.001**
Health facility (Primary)	566 (57.8)	444 (45.3)	**<0.001**
Medication pill burden	367 (37.6	243 (24.9)	**<0.001**
Recently acquired HIV^b^	232 (23.9)	232 (23.9)	1.00
Outcome and main predictors			
Past-week adherence	632 (64.5)	728 (74.3)	**<0.001**
Intimate partner violence past-year	34 (3.5)	29 (3.0)	0.52
Sexual abuse past-year	47 (5.0)	9 (1.0)	**<0.001**

SD, standard deviation. Bold signifies a statistically significant difference (*P* < 0.05).

a *P* value: chi-square test for the differences in reporting over time.

b The variable indicator for vertical/recent HIV acquisition was missing for (*n* = 9) participants.

### Viral suppression and adherence rates

A total of 64.5% of adolescents reported past-week ART adherence at wave 2 and 74.3% at wave 3. Only 51% reported past-week adherence at both time points. Self-reported adherence was associated with viral suppression (<50 copies/ml), controlling for age, sex, rural/urban location, poverty, time on ART treatment, vertical/recent HIV acquisition, and time point at both waves of data collection (*n* = 680, wave 2 and *n* = 597, wave 3) (see Table S4). A comparison of participants with any viral load record and those missing viral load showed no differences on most characteristics, except that participants with missing viral load were likely to be older (see Table S3).

### Associations of intimate partner violence and sexual abuse with adherence

Multivariable models (Table 2), adjusting for covariates, found that IPV was associated with lower odds of self-reported ART-adherence (aOR 0.41, 95% CI 0.21-0.78, *P* = 0.007). Sexual abuse was also associated with lower odds of self-reported ART-adherence (aOR 0.53, 95% CI 0.28–0.99, *P* = 0.048). A moderation model by sex (see Table S7 in Appendix) did not show any significant effect of male and female sex on the association of IPV and sexual abuse with adherence.

**Table 2:** Multivariable associations between intimate partner violence, sexual abuse, and past-week adherence to antiretroviral therapy among adolescents (*N* = 980 people, observations: 1960).

	Model 1 (full model)		Model 2 (*P* < 0.10)	
Factors	aOR (95% CI)	*P* value	aOR (95% CI)	*P* value
Sociodemographic factors						
Age	1.00 (0.95–1.05)	0.940		
Female	1.07 (0.84–1.36)	0.578		
Urban residence	**0.61 (0.46–0.82)**	**0.001**	**0.60 (0.45–0.80)**	**<0.001**
Poverty	**1.09 (1.03–1.15)**	**0.001**	**1.09 (1.03–1.14)**	**0.002**
Food insecurity	1.02 (0.91–1.14)	0.753		
In a relationship (currently)	1.03 (0.76–1.38)	0.866		
Caregiver biological parent	0.85 (0.67–1.07)	0.162		
Main predictors						
IPV past-year	**0.41 (0.21–0.79)**	**0.008**	**0.40 (0.21–0.78)**	**0.007**
Sexual abuse past-year	**0.52 (0.28–0.96)**	**0.038**	**0.53 (0.28–0.99)**	**0.048**
Other covariates						
Health facility (Community health centre)	**1.59 (1.04–2.44)**	**0.031**	**1.81 (1.26–2.61)**	**0.001**
Health facility (Hospital)	**1.51 (1.01–2.24)**	**0.042**	**1.74 (1.27–2.38)**	**0.001**
Health facility (Primary)	0.83 (0.61–1.14)	0.249		
Medication pill burden	**0.38 (0.30–0.49)**	**0.000**	**0.38 (0.30–0.49)**	**<0.001**
Recently acquired HIV^§^	**0.65 (0.47–0.91)**	**0.011**	**0.65 (0.50–0.86)**	**0.002**
Time point 3	**1.41 (1.12–1.78)**	**0.004**	**1.45 (1.16–1.81)**	**0.001**
Random part						
Individual variance $\sigma_{\mu }^{2}$ (SE)	0.47 (0.16)		0.51 (0.17)	
Inter-class correlation	0.11		0.13	

aOR, adjusted odds ratios; 95% CIs, 95% confidence intervals; SE, standard errors; these models adjusted for the time point of data collection as a time dummy variable. We checked for a correlation between IPV and sexual abuse and the items were not highly correlated. Bold signifies a statistically significant difference (*P* < 0.05).

§ The variable indicator for vertical/recent HIV acquisition was missing for (*n* = 9) participants.

**Fig. 1:**
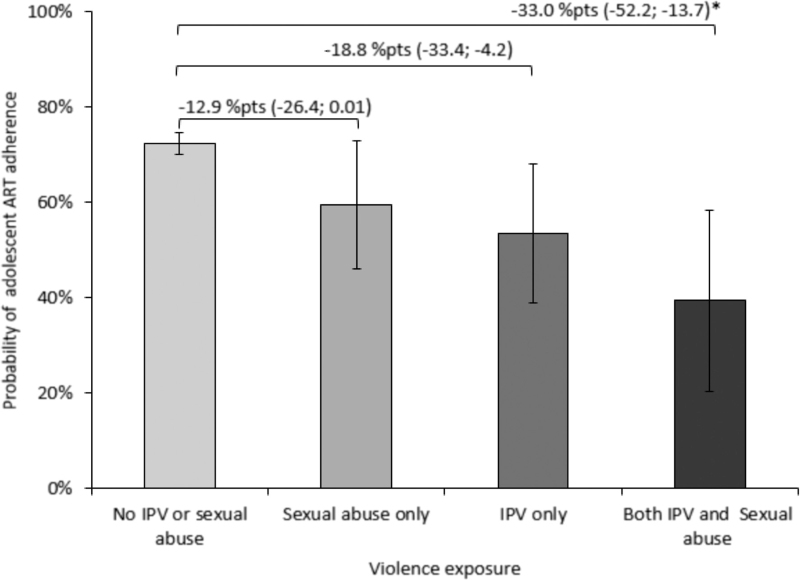
Adjusted predicted probabilities of past-week adherence.

Figure 1 shows the adjusted predicted probabilities and partial effects of adherence for the four possible combinations of IPV and sexual abuse. Adolescents with no sexual abuse or IPV exposure had a 72% probability of self-reported adherence (95% CI 70–74%). Those exposed to past-year sexual abuse had a 59% probability of adherence (95% CI 46–73%), while those exposed to past-year IPV had a 53% probability of adherence (95% CI 39–68%). Exposure to both past-year sexual abuse and IPV had a 39% probability of self-reported adherence (95% CI 20–58%) compared with no exposure, thus a minus 33% points difference (95% CI −52.2 to −13.7%).

Bars represent adjusted predicted probabilities of ART adherence for different combinations of key explanatory variables and their corresponding error bars (95% confidence intervals). ^∗^These are differences in the adjusted probabilities of ART adherence for each combination compared with a scenario where an adolescent is not experiencing both IPV and sexual abuse, and their corresponding 95% confidence intervals.

### Sensitivity analysis

We further carried out two types of sensitivity analysis. First, we assessed the primary predictors on self-reported past-week adherence for a sub-sample of those who reported being in partnerships (*N* = 403) at the time of data collection (see Table S8). Only IPV was associated with lower odds of self-reported adherence (aOR 0.33, 95% CI 0.16–0.69). Second, we modelled the primary predictors on the viral load as a biomarker, amongst a subset of participants with viral load measures. (see Table S9). In this subset, no primary predictors were associated with viral suppression (≤50 copies/ml) but non-significant associations were in the same direction as main analyses.

## Discussion

These findings show that exposure to sexual abuse and IPV is longitudinally associated with adolescent ART nonadherence. The adjusted probability of self-reported adherence was lower (38%) amongst those exposed to both forms of violence compared with 72% amongst nonvictimized adolescents. This study adds longitudinal and adolescent-specific evidence to previous cross-sectional studies in South Africa and Zambia –which suggested that sexual and IPV are important barriers to adolescent adherence. We note that the mean age of adolescents was 15 years, with only 30% in a relationship at Time 2 – so rates of IPV would likely rise rapidly in older adolescence.

We are at an important moment of opportunity to deliver on the 95–95–95 targets, but these goals may be impossible to achieve through services focused on HIV treatment and care alone. Our findings reflect high rates of violence reported across the region. These findings suggest an essential need for cross-sectoral collaboration between HIV care, violence prevention, and violence response services. Two recent reviews [40,41] identify increasing evidence for effective sexual violence prevention through parenting programmes, classroom, and community-based programmes [42,43] in the Sub-Saharan African region. There is also new evidence for social protection ‘cash plus’ models in preventing sexual violence, for example, livelihoods, life skills training, and a cash transfer in Tanzania [44]. Future research could valuably assess impacts of effective violence prevention on adolescent HIV treatment outcomes.

There is also an increasing evidence base for how we can integrate violence prevention in healthcare settings, including adolescent HIV services. Screening may be challenging because healthcare workers report time and resource overburden and hesitance to ask adolescents about sexual violence without having services to refer them to [45]. Whilst policies in the region have started to recognize the importance of responding to violence, there is often a lack of training or support for healthcare workers in implementing violence prevention strategies in healthcare settings. However, there are now good examples of screening tools, such as HEADSS and HEADSS+ [46], and effective approaches to linking adolescents living with HIV from clinic to community-based services, such as peer-supporter-led programmes and community workers [47–49]. There is strong evidence of the importance of access to postviolence care – both medical and psychosocial [50] – and further research should investigate how this may mitigate the impacts of violence on ART-adherence.

We note that this study has a number of limitations. First, we use self-reported adherence, which risks social desirability bias and recall error. We used recent (past-week) adherence to minimize recall bias and validated self-reported adherence against clinic-recorded viral suppression. Second, our younger and mixed-sex sample may have reduced power, as IPV and sexual abuse increases with age, and this could potentially lead to greater uncertainty around the effects of sexual abuse and IPV on adherence. However, the use of a repeated measures random-effects model meant more observations and increased statistical power. Third, this study focused on IPV in the past year, and did not measure sexual, technological, or financial IPV, leading to potential underestimation of IPV exposure. Fourth, because of our measurement approach, an adolescent who was exposed to sexual violence (but no other form of violence) by an intimate partner would fall into the 'sexual abuse’ category even though they were IPV-exposed. Fifth, this cohort had 3.4% mortality, with a risk that those adolescents who died were more vulnerable, again potentially risking underestimation of effects. Sixth, this study only included participants who had at some point initiated ART, and we recognize that those who never tested for HIV or accessed HIV care may have been even more vulnerable to the impacts of violence. Important strengths of the study include the total study area sampling and inclusion of adolescents no longer engaged in healthcare, which increases representativity of adolescents living with HIV, and adolescent co-design in measures and interviewing approach, which may have increased confidentiality and reduced discomfort in reporting. Longitudinal data allowed us to examine the joint influence of sexual abuse, IPV, and time on ART on adolescents’ ART adherence.

Sexual and IPV are strongly associated with reduced ART adherence among adolescents, affecting both girls and boys, and suggesting an urgent need to link violence prevention and health services for adolescents living with HIV. This will require availability of services at a local level, simple referral processes and accessibility for adolescents living with HIV, and improved reach by violence prevention services for ALHIV.

## Acknowledgements

We thank the South African National Health Laboratory Service (NHLS), and leadership by Prof Gayle Sherman, for provision of data and contribution to the conceptualisation of linking survey and NHLS data. E.T. and L.C. designed the overall study, including data collection tools. L.C. and S.Z. led the analyses and write-up of the manuscript with support from M.O. and wrote the manuscript draft. F.M., N.L., W.R., M.V., M.O., and E.T. provided edits and feedback on manuscript content and have approved the final draft. M.V. advised on focus and framing of research. All authors have reviewed and approved this manuscript. We are grateful to all study participants, their families and healthcare providers, who opened their hearts, minds, and personal/professional spaces to the study team, the Teen Advisory Group, and the research study team. We are additionally grateful to our teams at the Universities of Cape Town and Oxford.

Funding: The Mzantsi Wakho Study was supported by the Evidence for HIV Prevention in Southern Africa (EHPSA), a UK Aid programme managed by Mott MacDonald, Janssen Pharmaceutica N.V., part of the Janssen Pharmaceutical Companies of Johnson & Johnson, the Nuffield Foundation (CPF/41513; OPD/31598), but the views expressed are those of the authors and not necessarily those of the Foundation, the Regional Inter-Agency Task Team for Children Affected by AIDS – Eastern and Southern Africa (RIATT-ESA), the International AIDS Society through the CIPHER grant (155-Hod; 2018/625-TOS), but the views expressed in written materials or publications do not necessarily reflect the official policies of the International AIDS society, Claude Leon Foundation (F08559/C).

Support for the authors and team is provided by: the UKRI GCRF Accelerating Achievement for Africa's Adolescents Hub (Grant Ref: ES/S008101/1), the European Research Council (ERC) under the European Union's Horizon 2020 research and innovation programme (grant agreement n°771468), UNICEF Eastern and Southern Africa Office (UNICEF-ESARO), Oak Foundation (R46194/AA001 and OFIL-20-057), the Fogarty International Center, National Institute on Mental Health, National Institutes of Health under Award Number K43TW011434, and the Wellspring Philanthropic Fund. The content is solely the responsibility of the authors and does not represent the official views of the National Institutes of Health. This was also possible thanks to the UK Medical Research Council (MRC) and the UK Department for International Development (DFID) under the MRC/DFID Concordat agreement, and by the Department of Health Social Care (DHSC) through its National Institutes of Health Research (NIHR) (MR/R022372/1), University of Oxford's ESRC Impact Acceleration Account (IAA) (K1311-KEA-004 and 1602-KEA-189) and Clarendon-Green Templeton College Scholarship, the Economic and Social Research Council (IAA-MT13- 003), the John Fell Fund (103/757 and 161/033), the Leverhulme Trust (PLP-2014-095) and Research England.

### Conflicts of interest

There are no conflicts of interest.

## Supplementary Material

Supplemental Digital Content
