# Supplementary material for: Impacts of intimate partner violence and sexual abuse on antiretroviral adherence among adolescents living with HIV in South Africa
Source: AIDS. 2022 Dec 5;37(3):503–11. doi: 10.1097/QAD.0000000000003440 (PMC9894135; doi:10.1097/QAD.0000000000003440)
Supplement: Supplemental Digital Content [file aids-37-503-s001.docx]

# **Appendix**

**Table S1: Baseline characteristics for the full sample (N=1046)**

|  | **Past-week adherence at baseline** | | | |
| --- | --- | --- | --- | --- |
|  | **Total** | **No** | **Yes** |  |
|  | **(N=1046)** | **(N=365)** | **(N=681)** |  |
| **Baseline characteristics** | **N (%)** | **N (%)** | **N (%)** | **p-value** |
| **Socio-demographic factors** |  |  |  |  |
| Female | 576 (55.1) | 225 (61.64) | 351 (51.54) | **0.002** |
| Age (Mean/ SD) | 13.7 (2.89) | 14.19 (2.96) | 13.39 (2.82) | **<0.001** |
| Rural residence | 272 (26.0) | 115 (31.5) | 157 (23.1) | **0.003** |
| Food insecurity (days) (Mean/ SD) | 0.51 (1.29) | 0.71 (1.51) | 0.41 (1.15) | **<0.001** |
| Poverty (Mean/ SD) | 6.38 (1.80) | 6.17 (1.94) | 6.49 (1.71) | **0.006** |
| Caregiver biological parent | 468 (44.7) | 170 (46.58) | 298 (43.76) | 0.38 |
| In a relationship (currently) | 256 (24.5) | 116 (31.78) | 140 (20.56) | **<0.001** |
| **Outcome and main predictors** |  |  |  |  |
| Intimate partner violence past-year* | - | - | - |  |
| Sexual abuse past-year | 67 (6.41) | 27 ( 7.40) | 40 ( 5.87) | 0.34 |
| **Other covariates** |  |  |  |  |
| Health facility (Community health centre) | 144 (13.8) | 52 (14.25) | 92 (13.51) | 0.74 |
| Health facility (Hospital) | 469 (44.8) | 135 (36.99) | 334 (49.05) | **<0.001** |
| Health facility (Primary) | 165 (15.8) | 70 (19.18) | 95 (13.95) | **0.027** |
| **HIV-related factors** |  |  |  |  |
| Recently acquired HIV | 222 (21.2) | 102 (28.65) | 120 (17.67) | **<0.001** |
| Medication pill burden | 361 (34.5) | 149 (40.82) | 212 (31.13) | **0.002** |
| Viral suppression (≤ 50 copies/mL)^x^ | 519 (62.5) | 148 (56.70) | 371 (65.09) | **0.021** |

*IPV was not measured at the baseline of the main study; ^§^ The variable indicator for vertical/recent HIV acquisition was missing for (n=11) participants at baseline; ^x^ Viral load was available for (N=831 participants at baseline of the main study).

**Table S2: Factors associated with loss-to-study follow-up between the two-time points (n=1030)**

|  | **Complete**  **(N=980)** | **Lost-to-study follow-up**  **(N=50)** |  |
| --- | --- | --- | --- |
| **Socio-demographic factors** | **N (%)** | **N (%)** | **p-value** |
| Age | 15.25 (3.03) | 16.70 (3.03) | **<0.001** |
| Female | 556 (57) | 29 (58) | 0.86 |
| Rural residence | 244 (25) | 12 (24) | 0.95 |
| Poverty (Mean /SD) | 5.52 (2.28) | 5.74 (2.04) | 0.50 |
| Recently acquired HIV | 232 (24) | 10 (20) | 0.53 |

**Table S3: Multivariate factors associated with missing viral load (VL) – (n=970 people- 1940 observations)**

|  | **Missing VL** | |
| --- | --- | --- |
| **Factors** | **aOR (95% CI)** | **p-value** |
| Age | 1.12 (1.05-1.19) | **0.001** |
| Female | 0.97 (0.69-1.37) | 0.874 |
| Urban residence | 1.20 (0.83-1.74) | 0.337 |
| Poverty | 0.96 (0.90-1.02) | 0.219 |
| Recently acquired HIV | 1.30 (0.81-2.09) | 0.280 |
| Time on ART treatment* | 0.95 (0.90-1.00) | 0.055 |

*Time on treatment was measured in years since ART initiation. N=300 and N=383 participants were missing VL at both time points- respectively**.**

**Table S4: Association between past-week adherence and non-detectable viral load (n=785 people- 1273 observations)**

|  | **VL suppression (≤ 50 copies/mL)** | |
| --- | --- | --- |
| **Factors** | **aOR (95% CI)** | **p-value** |
| Past-week adherence | 1.81 (1.21-2.71) | **0.004** |
| Age | 0.89 (0.82-0.97) | **0.008** |
| Female | 1.43 (0.93-2.20) | 0.102 |
| Urban residence | 1.94 (1.20-3.12) | **0.007** |
| Poverty | 1.03 (0.95-1.11) | 0.481 |
| Recently acquired HIV | 1.06 (0.55-2.06) | 0.862 |
| Time on ART treatment | 1.07 (1.00-1.15) | **0.036** |
| Time point | 0.54 (0.38-0.75) | **<0.001** |

^*^aOR adjusted odds ratio

**Table S5: Wald test for the significance of separating within-between effects**

| **Equation 1: Multivariable Hybrid model** | | **Wald test** | |
| --- | --- | --- | --- |
| **Factors** | **aOR (95% CI)** | $\chi^{2}$ **value** | **p-value** |
| IPV_Between_ | 0.34 (0.13-0.92) | 0.31 | 0.578 |
| IPV_within_ | 0.49 (0.21-1.16) |  |  |
| Sexual Abuse_Between_ | 0.41 (0.15-1.13) | 0.50 | 0.478 |
| Sexual Abuse_Within_ | 0.67 (0.28-1.57) |  |  |

^*^Model adjusted for all covariates in Table 1

**Table S6: Goodness of fit statistics: Akaike's information criterion and Bayesian information criterion**

| **Model** | **N** | **Log likelihood (model)** | **df** | **AIC** | **BIC** |
| --- | --- | --- | --- | --- | --- |
| **RE** | **1913** | **-1086.5** | **17** | **2207.087** | **2301.55** |
| REWB_IPV | 1913 | -1082.7 | 26 | 2217.398 | 2361.87 |
| REWB_SAbuse | 1913 | -1084.4 | 26 | 2220.733 | 2365.20 |
| REWB_Full | 1913 | -1080.6 | 28 | 2217.11 | 2372.69 |
| REWB_IPV-Hybrid model with intimate partner violence as the main predictor; REWB_SAbuse-Hybrid model with sexual abuse as the main predictor; REWB_Full-Hybrid model with both intimate partner violence and sexual abuse as the main predictors; RE-random effects model | | | | | |

**Table S7: Multivariable associations between IPV, sexual abuse and adherence to ART: moderation model by sex (N=980; observations 1960)**

|  | **Past-week adherence** | |
| --- | --- | --- |
| **Variables** | **aOR (95% CIs)** | **p-value** |
| **Socio-demographic factors** |  |  |
| Rural | **1.66 (1.25-2.21)** | **0.001** |
| Age | 1.06 (0.83-1.35) | 0.659 |
| Poverty | **1.08 (1.03-1.14)** | **0.001** |
| Medication pill burden | **0.38 (0.30-0.49)** | **<0.001** |
| Health facility Hospital | **1.74 (1.28-2.37)** | **<0.001** |
| Health facility CHC | **1.81 (1.24-2.64)** | **0.002** |
| Recent acquired HIV | **0.64 (0.49-0.85)** | **0.002** |
| **Main predictors** |  |  |
| Sexual abuse | 0.43 (0.14-1.34) | 0.144 |
| Intimate partner violence (IPV) | 0.42 (0.12-1.41) | 0.160 |
| **Interaction terms** |  |  |
| Female # Sexual abuse | 1.34 (0.35-5.18) | 0.671 |
| Female # IPV | 0.94 (0.23-3.83) | 0.931 |
| Time point 3 | **1.45 (1.16-1.81)** | **0.001** |
| **Random part** |  |  |
| Individual variance $\sigma_{\mu}^{2}$ (SE) | 0.52 (0.16) | |
| $\rho$ Inter-class correlation | 0.143 | |

^*^aOR adjusted odds ratio; 95% CI- 95% confidence intervals; SE- standard errors; These models adjusted for the time point of data collection as a time dummy variable. CHC- community health centre.

**Table S8: Sensitivity analysis of the primary predictors among only those who report being partnered in the past year (N=403; observations 605)**

|  | **Past-week adherence** | |
| --- | --- | --- |
| **Variables** | **aOR (95% CI)** | **p-value** |
| **Socio-demographic factors** |  |  |
| Age | 1.06 (0.96-1.18) | 0.226 |
| Female | 1.03 (0.65-1.64) | 0.892 |
| Urban residence | 0.82 (0.51-1.32) | 0.416 |
| Poverty | 1.09 (1.00-1.19) | 0.051 |
| Food insecurity | 0.98 (0.81-1.18) | 0.819 |
| In a relationship (currently) |  |  |
| Caregiver biological parent | 1.01 (0.68-1.51) | 0.946 |
| **Main predictors** |  |  |
| IPV past-year | **0.33 (0.16-0.69)** | **0.003** |
| Sexual abuse past-year | 0.49 (0.18-1.32) | 0.156 |
| **Other covariates** |  |  |
| Health facility CHC | **2.55 (1.19-5.47)** | **0.016** |
| Health facility Hospital | 1.65 (0.77-3.52) | 0.195 |
| Health facility Primary | 1.13 (0.68-1.90) | 0.637 |
| Medication pill burden | **0.27 (0.17-0.43)** | **<0.001** |
| Recently acquired HIV | **0.51 (0.31-0.84)** | **0.008** |
| Time point 3 | 1.03 (0.68-1.55) | 0.889 |
| **Random part** |  |  |
| Individual variance $\sigma_{\mu}^{2}$ SE | 0.29 (0.16) |  |
| $\rho$ Inter-class correlation | 0.13 |  |

^*^aOR adjusted odds ratio; 95% CI- 95% confidence intervals; SE- standard errors; These models adjusted for the time point of data collection as a time dummy variable. CHC- community health centre.

**Table S9: Sensitivity analysis of the primary predictors among only those with viral load (N=784; observations 1266).**

|  | **Viral suppression (≤ 50 copies/mL)** | |
| --- | --- | --- |
| **Variables** | **aOR 95% CI** | **p-value** |
| **Socio-demographic factors** |  |  |
| Age | **0.90 (0.82-0.98)** | **0.015** |
| Female | 1.46 (0.97-2.20) | 0.068 |
| Urban residence | **1.79 (1.12-2.84)** | **0.014** |
| Poverty | 1.03 (0.96-1.12) | 0.378 |
| Food insecurity | **0.80 (0.66-0.97)** | **0.024** |
| In a relationship (currently) | 0.98 (0.64-1.51) | 0.920 |
| Caregiver biological parent | 0.98 (0.66-1.46) | 0.939 |
| Time on ART treatment | **1.08 (1.01-1.15)** | **0.019** |
| **Main predictors** |  |  |
| IPV past-year | 0.56 (0.21-1.52) | 0.255 |
| Sexual abuse past-year | 0.71 (0.26-1.91) | 0.494 |
| **Other covariates** |  |  |
| Health facility CHC | 0.86 (0.43-1.73) | 0.672 |
| Health facility Hospital | 0.75 (0.42-1.35) | 0.332 |
| Health facility Primary | 1.01 (0.60-1.69) | 0.981 |
| Medication pill burden | **0.54 (0.37-0.79)** | **0.002** |
| Recently acquired HIV | 1.09 (0.58-2.05) | 0.796 |
| Time point 3 | **0.50 (0.35-0.69)** | **<0.001** |
| **Random part** |  |  |
| Individual variance $\sigma_{\mu}^{2}$ SE | 0.24 (0.14) | |
| $\rho$ Inter-class correlation | 0.49 | |

^*^aOR adjusted odds ratio; 95% CI- 95% confidence intervals; SE- standard errors; These models adjusted for the time point of data collection as a time dummy variable. CHC- community health centre.
